# Cross-sectional associations between overweight, eating behavior, and physical activity in children and adolescents: differences depending on socio-economic status

**DOI:** 10.1186/s40795-025-01230-y

**Published:** 2026-01-07

**Authors:** Tanja Poulain, Peggy Ober, Charlotte Kühnelt, Ulrike Spielau, Carolin Sobek, Wieland Kiess, Tobias Lipek

**Affiliations:** 1 LIFE Child Leipzig Research Center for Civilization Diseases, Leipzig University, Leipzig, Germany; 2 Department of Women and Child Health, Hospital for Children and Adolescents, Center for Pediatric Research (CPL), Leipzig University, Leipzig, Germany; 3 Integrated Research and Treatment Center Adiposity Diseases, Medical Faculty, Leipzig University, Leipzig, Germany; 4 German Center for Child and Adolescent Health (DZKJ), partner site Leipzig/Dresden, Leipzig, Germany; 5 Department of Epidemiology and Health Monitoring, Robert Koch Institute, Berlin, Germany

**Keywords:** Overweight, Diet, Physical activity, Children, Socio-economic status

## Abstract

**Background:**

This study assesses associations between eating behavior and physical activity (PA) and overweight/obesity in children and adolescents, focusing on differences depending on familial socio-economic status (SES).

**Methods:**

Data were collected within a school-based study. The sample comprised 661 8- to 15-year-old children and adolescents from families with either low (*n* = 77), medium (*n* = 367), or high (*n* = 217) SES. Overweight, including obesity, was defined as a Body Mass Index (BMI) standard deviation score above the 90th percentile. Eating behavior was assessed using the parent-version of the Composition and Culture of Eating Questionnaire (CoCu). Regarding PA, we compared children performing versus not performing any PA in their leisure time. Logistic regression analyses were applied to assess associations between overweight and healthiness of diet, culture of eating (media use while eating and snacking between meals), and leisure PA. All associations were checked for interactions with SES.

**Results:**

The prevalence of overweight was 22% in low SES families, 14% in medium SES families, and 6% in high SES families. Overweight was significantly associated with a less healthy diet, but this association was only shown in children from families with medium SES. Media use while eating and snacking between meals were more frequent in children with overweight, while PA was less frequent. Family SES did not moderate the strengths of these associations.

**Conclusions:**

Unhealthier diet and eating habits as well as less PA are associated with overweight in children and adolescents. However, associations with healthy diets were not observed in families with low or high SES, suggesting that other factors may play a greater role in these groups.

## Background

In developed countries, the prevalence of overweight and obesity in children and adolescents has increased in the last decades [1] and reached a plateau at a high level or even decreased in recent years [2, 3]. In Germany, the prevalence of overweight, including obesity, in children aged 3 to 17 years was 15% in 2014–2017, and the prevalence of obesity was 6% [4]. Many individuals who are overweight in early childhood stay overweight during adolescence or even become obese [5]. Furthermore, overweight and obesity represent a major health risk, as they increase the risk of other health problems such as metabolic and cardiovascular diseases [6, 7].

Weight development is shaped by several factors, including genetic, environmental (e.g., food environment), social (e.g., familial education and income, eating habits at home), and behavioral factors [8]. In terms of behavioral factors, eating behavior and physical activity (PA) are among the most decisive factors. According to the energy balance model of obesity, differences between energy intake and energy expenditure due to an unhealthy diet and lack of physical activity are directly linked to weight gain [9].

In the context of eating behavior, the dietary composition as well as the culture of eating might impact weight development. Diet refers to what children eat. In developed countries, where the availability of fatty, sweet, and ultra-processed food is high [10], a high consumption of these foods has been shown to be associated with higher body weight [11–13]. Culture of eating, on the other hand, refers to meal-related behaviors such as eating together as a family, using media during meals, or snacking between meals [14]. In Western societies, distraction by electronic media [15–18], eating alone [15, 18, 19], and snacking unhealthy foods between meals [20, 21] are associated with increased weight.

Children’s PA has also been shown to be associated with children’s weight status [22, 23]. In more recent studies conducted in Germany, overweight or a higher Body Mass Index (BMI) in primary school children occurred significantly more frequently when the children were less physically active [17, 24]. A previous review underlines the important role of school-based PA and healthy diets in preventing overweight and obesity in children [25].

With respect to social factors, several studies showed an association between a lower socio-economic status (SES) and higher BMI or a higher prevalence of overweight and obesity in children, at least in developed countries [26], including Germany [13, 27, 28]. This association might be explained by unhealthier eating habits, more sedentary behavior, and less healthy diets in children from families with low SES [13, 28–30]. In addition to associations between SES and health indicators, some studies suggest that associations between health behaviors and health indicators might differ depending on SES. For example, in own preliminary work, overweight/obesity in one- to 14-year-old children was associated with shorter sleep duration, but this association was only significant in low-SES families [31]. In another study, a higher BMI in four- to 10-year-old children was associated with fewer behavioral difficulties in low-SES families, but not in medium-SES families [32].

The present analysis investigated associations of eating behavior (composition of diet and culture of eating) and PA with overweight/obesity (hereinafter referred to as overweight) in children and adolescents, with a specific focus on differences between socio-economic strata. We expected to observe a less healthy diet, poorer eating habits, and lower PA in children with overweight and in children from families with low SES. Furthermore, we expected the associations to be stronger in families with low or medium SES than in families with high SES.

## Methods

### Participants

Data for the present project were collected between 2018 and 2019 within the cross-sectional Leipzig School Nutrition Study conducted at elementary and secondary schools in 15 of the total of 63 city districts in the city of Leipzig, Germany [33, 34]. These districts were chosen based on the prevalence of low SES and childhood overweight (including obesity). Twelve districts were characterized by a rather low neighborhood SES (percentage of people receiving basic income support > 15%) and a high prevalence of overweight (> 10% in school entrance examination), while three districts were characterized by high neighborhood SES (percentage of people receiving basic income support < 5%) and low overweight prevalence (< 4% in school entrance examination) [35–37]. In 2018, about 30% of the total population of Leipzig lived in these areas [38]. Of 42 eligible schools, 34 consented to participate; 24 primary schools and 14 secondary schools. Four schools included both primary and secondary school types. Before the children participated, the study coordinator explained the study procedure to them and gave them the parental consent form to take home. The completed consent forms were handed in at school. Only children whose parents had provided informed written consent were able to participate.

**Fig. 1:**
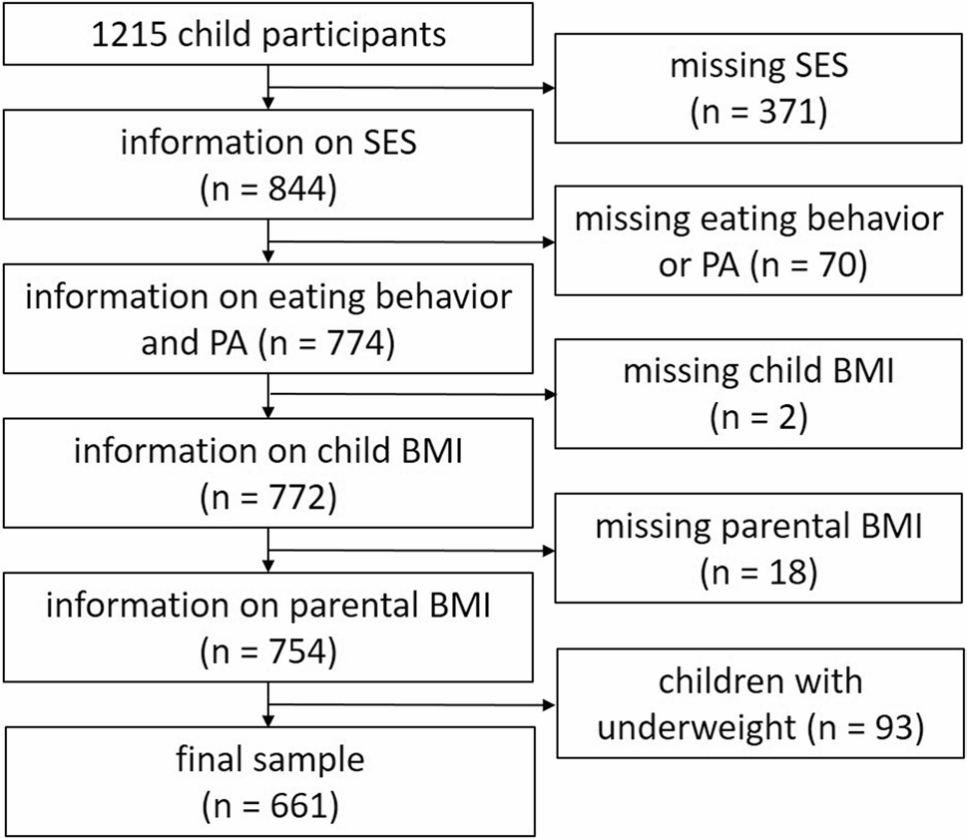
Overview of included and excluded study participants

A total of 1215 children participated in the Leipzig School Nutrition Study. For the present analysis, only participants with complete data on SES (371 missing values), diet and PA (70 further missing values), height and body weight/BMI of children (2 further missing values), and BMI of parents (18 further missing values) were eligible. In order to avoid distortions caused by possible changes in dietary habits and physical activity in underweight children, we also excluded children who were underweight (BMI-SDS < 10th percentile, *n* = 93). The final sample comprized 661 children aged 8 to 15 years (334 boys and 327 girls), as shown in Fig. 1. The high number of drop-outs can mainly be explained by the fact that some parents did not complete the parental questionnaire or did not provide any information on their education or income/SES.

Participants in primary school (*n* = 385, 50% boys) were in grade 4 (mean age = 10.2 years, sd = 0.54). Participants in secondary schools (*n* = 276, 51% boys) were in grades 6 to 8 (mean age = 12.6 years, sd = 0.73). In the study region, two types of secondary schools exist: lower secondary schools (grades 5 to 10, leading to a school degree not allowing access to university) and upper secondary schools (grades 5 to 12, leading to a school degree allowing access to university). In the Leipzig School Nutrition Study, 7 lower secondary schools (*n* = 79, 63% boys) and 7 upper secondary schools (*n* = 197, 48% boys) participated.

The study was approved by the Ethics Committee at the Medical Faculty of Leipzig University (483/17-ek).

### Data collection procedure

The study was mainly conducted in the classrooms of the participating schools and contained several measurements and tests. In the present analysis, only the measurements of body weight and height in order to calculate children’s BMI were used for further analyses. Information on children’s eating behavior, their PA, parents’ BMI, the family’s SES, and children’s migration background were assessed using a parental questionnaire completed at home, after the investigation at school. The parental questionnaires were returned to school by the children, where they were collected by the research team. At the end of the study, each child received an incentive of 5 euros.

### Measures

#### BMI

During the process of weighting and measuring, children wore underwear and one layer of clothing. They did not wear shoes. Body height was measured with an accuracy of 0.1 cm, using a portable stadiometer (Seca, Hamburg, Germany), and body weight was measured with an accuracy of 0.1 kg using a calibrated electronic scale (Kern, Balingen, Germany). Body weight was corrected for the estimated weight of the clothing [39, 40].

The BMI was calculated and transformed to standard deviation scores (SDS) using sex- and age-specific German references [41]. Based on the German guidelines of the German Working Group of Obesity in Childhood and Adolescents [42], a BMI-SDS of > 1.28 (> 90th percentile) was considered as overweight (including obesity, defined as BMI-SDS > 97th percentile). All BMI-SDS < 1.28 were considered normal weight. Children with underweight (BMI-SDS < −1.28, < 10th percentile) were excluded from the analysis.

#### Children’s eating behavior

Eating behavior of children was assessed using the parent version of the Composition and Culture of Eating (CoCu) screening questionnaire [14]. This questionnaire consists of two parts, the diet composition part and the culture of eating part. The diet composition part comprises 14 items assessing how many portions per day or week (ranging from 0 to 7 or more) children eat of different foods, e.g., fruits/vegetables, unsweetened dairy products, sweetened drinks. The responses are transformed into a Nutritional Health Score (NHS) ranging from − 120 to + 120. For further analysis, this score was divided by 10, i.e., the final score ranged from − 12 to + 12, with higher scores indicating healthier diets.

The culture of eating part of the questionnaire contains several questions about how children eat. Here, the questions on whether or not they use electronic media while eating (“Is the TV usually running at home during dinner, or is a tablet, smartphone, cell phone, or similar being used?”) and whether or not they snack unhealthy food between meals (“Does your child usually snack between meals, e.g., chocolate, gummybears, potato chips?”) were analyzed. Both questions could be answered with yes or no (reference = no). Reliability (re-test reliability) and validity (correlation with a Food Frequency Questionnaire) of the CoCu were shown previously [14]. Due to some missing data for these two items, the specific analyses were performed on slightly smaller samples (*n* = 658 for media use while eating and 646 for snacking between meals).

#### Leisure physical activity (PA)

Leisure PA was assessed by asking parents to indicate whether or not their children participate in organized PA (“Does your child play sports in a club?”) or non-organized PA (“Does your child play sports outside a club?”) in their leisure time/outside school. Responses to the two questions were combined and children were categorized as either performing any leisure PA or not (reference = performing PA). The questionnaire was originally designed for the use in the LIFE Child study, a large cohort study on child development [43]. So far, validity and reliability of the instrument were not assessed.

#### SES, migration background, and parental BMI

To assess the family’s SES, parents provided information on their school and professional education (ranging from no school degree to University degree), their current occupation, and their monthly equivalized disposable household income. The exact questions and response options are reported elsewhere [44]. For each parameter, a score ranging from 1 (lowest) to 7 (highest) is calculated. These scores are combined to an SES composite score ranging from 3 to 21, with higher scores indicating higher SES [44]. Based on cut-offs created in a representative German study, the SES score can be used to classify a family’s SES as either low (scores 3–8.4.4), medium (scores 8.5–15.5), or high (scores 15.5–21) [44].

Regarding migration background, we asked parents whether they were born in Germany. If at least one parent was not born in Germany, the child was considered to have a migration background.

For the assessment of parental BMI, parents were asked to indicate their body weight and their height. Due to the high number of missing values for body weight and height of the fathers (*n* = 85), we mainly used the data of the mothers (*n* = 649). In 12 cases, no information was available for mothers, but for fathers. The strength of the analyzed associations did not differ depending on whether the paternal BMI was considered or the corresponding children were excluded. Therefore, we decided to consider paternal BMI instead of maternal BMI for these children.

### Statistical analysis

Study data were collected and managed using REDCap electronic data capture tool [45, 46]. Statistical analysis was performed using R Version 4.4.0 [47]. Sample characteristics were described in terms of means and standard deviations or numbers and percentages. We performed t-tests and chi-squared tests to compare age, sex, BMI-SDS, and school type of children included or excluded from analysis. Differences in variables depending on SES (low, medium, high) were assessed using ANOVAs or chi-squared tests. For the assessment of associations between eating behavior or leisure PA (independent variables) and overweight (dependent variable), we first applied logistic regression analyses within the whole sample. In a second step, we investigated whether the independent variables interacted with the SES, i.e., whether strengths of associations differed depending on SES. If an interaction was statistically significant (*p* <.05), the according association was assessed in each SES group separately. As participants were nested within schools, the school of each participant was included as random factor in each model. In addition, all associations were adjusted for age, sex, parental BMI, and type of school. Only in the single analyses in the high SES group, type of school was not included as covariate as the number of children from lower secondary school was < 5% in this group. Associations were considered statistically significant if the p-value was < 0.05.

## Results

### Socio-demographic characteristics, body weight, eating behavior, and leisure PA in the present sample

The final sample included 661 children. Characteristics of the sample are shown in Table 1. Compared to children excluded from the analysis (*n* = 554), included children, on average, were significantly younger (11.2 years versus 11.4 years), had a higher BMI-SDS (0.11 versus − 0.23), and less frequently attended a lower secondary school (11% versus 18%, all *p* <.05).

**Table 1:** Characteristics of the study sample by family SES

	*n*		Total sample *n* = 661	Low SES *n* = 77	Medium SES *n* = 367	High SES *n* = 217
Socio-demographics						
Sex	661	n (%) female	327 (49%)	37 (52%)	180 (51%)	100 (46%)
Age	661	mean (sd)	11.22 (1.30)	11.30 (1.46)	11.19 (1.31)	11.23 (1.25)
School type***	661	n (%) primary	385 (58%)	56 (73%)	213 (58%)	116 (53%)
		n (%) upper secondary	197 (30%)	6 (8%)	99 (27%)	92 (42%)
		n (%) lower secondary	79 (12%)	15 (19%)	55 (15%)	9 (4%)
Migration background	645	n (%) yes	99 (15%)	14 (18%)	57 (15%)	28 (12%)
Weight characteristics						
Overweight***	661	n (%) yes	81 (12%)	17 (22%)	50 (14%)	14 (6%)
BMI-SDS***	661	mean (sd)	0.11 (0.91)	0.43 (1.03)	0.11 (0.94)	0.01 (0.80)
Parental BMI***	661	mean (sd)	24.71 (5.32)	27.38 (6.40)	24.90 (5.54)	23.43 (3.99)
Eating behavior						
NHS***	661	mean (sd)	3.39 (2.88)	1.77 (3.10)	3.22 (2.71)	4.25 (2.80)
Media use while eating***	658	n (%) yes	143 (22%)	35 (45%)	88 (24%)	20 (9%)
Snacking between meals***	646	n (%) yes	389 (60%)	60 (80%)	218 (59%)	111 (51%)
Physical activity						
Leisure PA***	661	n (%) yes	524 (79%)	41 (53%)	288 (78%)	195 (90%)

Overweight was defined as a BMI-SDS > 90th percentile and, therefore, included obesity

*SES* Socio-economic status, *NHS* Nutritional Health Score, *PA* Physical activity, *BMI-SDS* Body mass index standard deviation score

*** statistically significant (*p* <.001) difference between SES groups

Regarding SES differences within the final sample, the distribution of sex, age, and migration background did not differ depending on SES, while the distribution of school type and variables related to children’s or parents’ body weight, children’s diet or leisure PA did. With respect to school type, the number of children from lower secondary schools decreased with increasing SES, while the number of children from upper secondary schools increased. BMI of parents, BMI-SDS of children, and the prevalence of overweight in children decreased with increasing SES. With respect to diet, children from families with low SES reported lower nutritional health, more frequent media use while eating, and more snacking between meals than children from families with medium or higher SES. Leisure PA, in contrast, was significantly less frequent in children from low SES families.

### Associations of eating behavior and PA with overweight

**Fig. 2:**
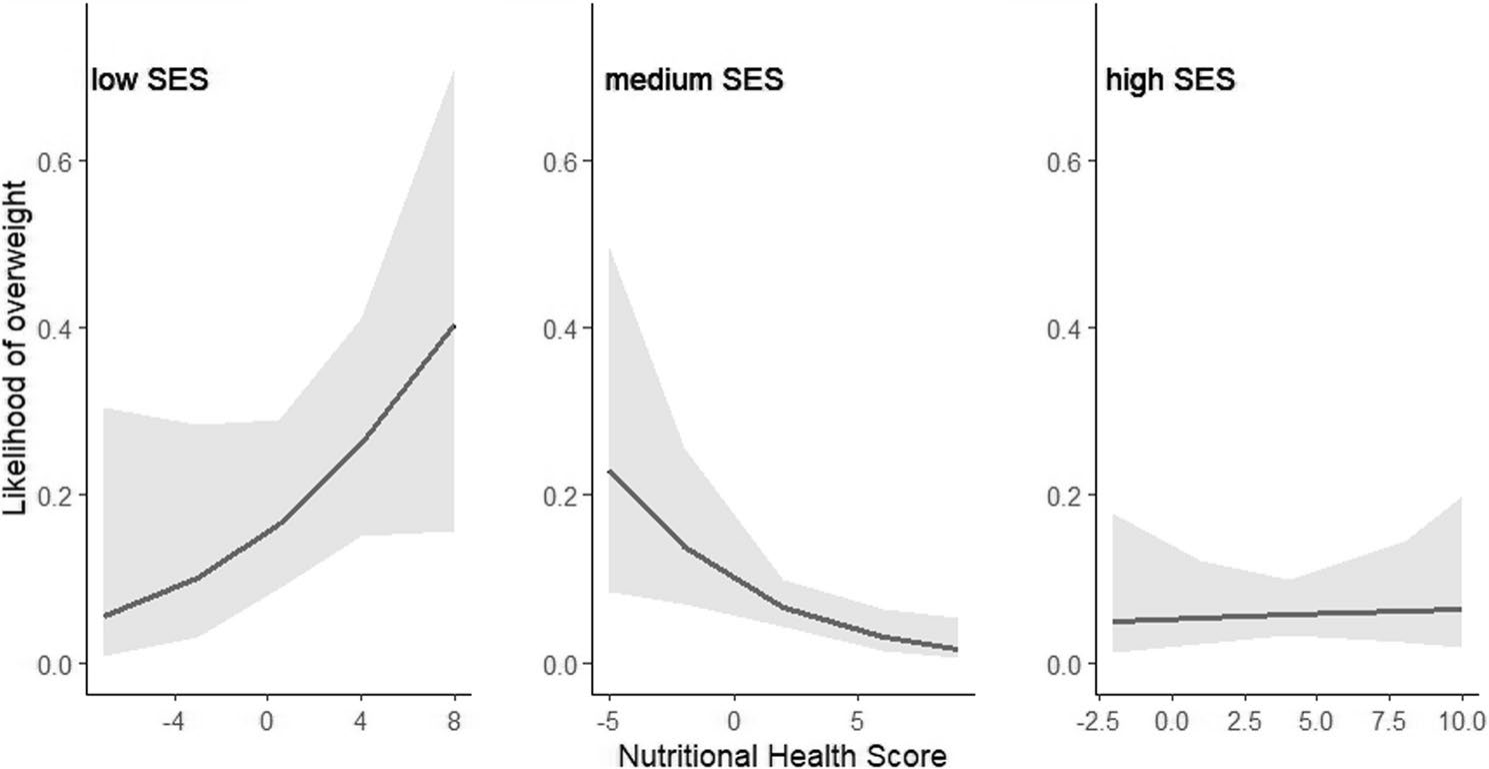
Effect plots illustrating the association (+ 95% confidence interval) between NHS (Nutritional Health Score) and overweight (including obesity) in children from families with low, medium, or high SES (socio-economic status)

The analyses revealed a significant negative association between the NHS and overweight (OR = 0.92, 95% CI 0.84–0.99, *p* =.026), i.e., a healthier diet was associated with a lower prevalence of overweight in the whole sample, as expected. Interestingly, a significant interaction between NHS and SES (*p* <.001) revealed differences in the strengths of this association depending on the family SES. In separate analyses, the negative association between NHS and overweight was only confirmed in children from families with a medium SES (OR = 0.81, 95% CI 0.69–0.95, *p* =.010). In this group, the estimated percentage of overweight was < 1% for children with a high NHS of + 8 but 14% in children with a low NHS of −2 (see Fig. 2). In contrast, in children from families with a low SES, the prevalence of overweight increased with increasing NHS (OR = 1.18, 95%CI 0.96–1.45), contrary to our hypothesis. However, the amount of uncertainty in this group was very large (see Fig. 2) and the association was not statistically significant (*p* =.117). In children from families with a high SES, the likelihood of being overweight was generally low and not significantly associated with the NHS (OR = 1.02, 95%CI 0.84–1.25, *p* =.833, see Fig. 2).

Similar to a lower NHS, media use while eating, snacking between meals, and not performing any PA were significantly associated with a higher prevalence of overweight in the whole sample (see Table 2). Regarding media use while eating, the estimated prevalence of overweight was 25% when children used media while eating, compared to 8% when they did not. Concerning snacking, the estimated likelihood of overweight was 13% in children reporting regular snacking but only 8% in children not snacking between meals. With respect to PA, 17% of children not performing any PA were estimated to be overweight, compared to 10% of children being physically active in their leisure time. In contrast to the association between NHS and overweight, the associations of media use while eating, snacking between meals, and PA with overweight were not significantly moderated by the family SES, i.e., the strengths of these associations did not differ depending on SES.

**Table 2:** Associations between culture of eating, PA, and overweight (*n* = 661)^a^

Independent variables		Overweight
Media use while eating	OR (95% CI)	4.01 (2.43–6.63)
	p	< 0.001
Snacking between meals	OR (95% CI)	1.82 (1.05–3.15)
	p	0.033
No leisure PA	OR (95% CI)	1.82 (1.05–3.17)
	p	0.032

Overweight was defined as a BMI-SDS > 90th percentile and, therefore, included obesity. All associations were adjusted for age, sex, and parental BMI

*PA* Physical activity

^a^for media use while eating and snacking between meals, the sample sizes were slightly smaller due to missing values (*n* = 658 and 646, respectively)

## Discussion

This study assessed associations between eating behavior and leisure PA and overweight in children and adolescents participating in a school-based study. Overall, the nutritional health of children in the present study was comparable with data shown in a larger sample of children in the same city [14]. Regarding leisure PA, 21% of children and adolescents were not physically active in their leisure time. This finding is concerning and in line with other studies highlighting the insufficient PA in children and youth [48], also in Germany [49]. One main finding of our study is that eating behavior, leisure PA, and the prevalence of overweight varied across SES-groups. Children from families with a high SES showed healthier eating habits, were more likely to be physically active, and were less frequently overweight than children from families with medium or low SES, with the latter group only insufficiently represented in our study. This finding aligns with previous studies in German children [13, 28–30] and shows that social inequality can already be observed in childhood and adolescence and may affect several areas of behavior and health. Another main finding is that associations between eating behavior and overweight varied depending on the family’s SES.

### Associations between diet composition and overweight

As hypothesized, the associations between an unhealthy composition of diet and overweight was statistically significant in the whole sample. Also in line with our expectations, the strength of this association differed depending on the SES of the family. In the largest group of children with a medium SES, we observed the expected association. In this group, overweight was more frequent when the diet of children was reported to be less healthy. This finding strengthens the assumption that a less healthy diet is a risk factor for overweight in childhood as it creates an imbalance of energy [9].

In contrast, in children from families with a low SES, overweight was not significantly associated with diet. The association even pointed in the unexpected direction. Based on previous studies showing stronger associations between health behavior and health indicators in children from families with low SES [31, 32], we had expected to find an especially strong association between unhealthy diet and overweight in these children. Importantly, the finding of a (not statistically significant) association between healthier diet and higher prevalence of overweight should generally be interpreted with caution due to the relatively small number of children from families with low SES in our sample. Another possible explanation for the finding is that parents with low SES whose children are overweight might show a stronger reporting bias, i.e., a stronger tendency to respond in a socially desired way, than parents from higher SES and parents whose children are normal-weight. Another study has already shown that biases in dietary reporting are stronger in people with low SES [50].

In children from families with a high SES, overweight was not associated with diet composition. The generally low prevalence of overweight in this group (6%) might be a main reason for this finding. Also, other factors like genetic predispositions might play a more essential role in this group.

### Associations between culture of eating and overweight

As expected, the analyses revealed significant associations between a better culture of eating and a lower prevalence of overweight. In more detail, the overweight prevalence was lower in children not using media while eating and in children not snacking between meals. In contrast to the composition of diet, the strengths of these associations were comparable across SES strata.

Overall, these findings are in line with previous studies [15–18] and emphasize the importance of the eating culture with regard to overweight in children. Media use while eating might distract children, which could lead to children eating too much and not realizing when they are full [15, 17]. Additionally, a potential lack of social interaction during meals due to distraction by electronic media might make it difficult to form healthy eating habits [15, 17]. Snacking between meals can increase the consumption of nutritionally unfavorable foods throughout the day. Also, snacking has been shown to have poor satiating efficiency [51].

### Associations between leisure PA and overweight

Children not being physically active in their leisure time were significantly more likely to be overweight than children being physically active, as expected. As for eating culture, the strength of this association did not differ between different SES groups. These findings suggest that PA may play a role in the development or maintenance of overweight in children and adolescents in all social strata. This confirms the assumption that weight gain is related to an imbalance between energy intake and expenditure [9]. It is also in line with previous reviews [22, 23, 25] and studies conducted in Germany [17, 24]. One review concludes that (school-based) interventions with combined diet and physical activity components are most effective in preventing overweight or obesity in children [25].

### Strengths and limitations

This analysis assessed associations between PA and eating behavior, and overweight in school-aged children in Germany. Strengths are the consideration of different aspects of eating behavior, namely diet composition and culture of eating, the large sample size, and the separate analyses for different social strata. However, the analysis has some limitations. One limitation is the under-representation of children from families with low SES (about 10%) compared to children from families with high SES (about 30%), resulting in limited generalizability. The comparison between included and excluded children furthermore indicated a selection bias in favor of children from upper secondary schools. Due to large amount of missing values, we were not able to include both maternal AND paternal BMI as covariates. In addition, PA, diet, and culture of eating were assessed via parental questionnaires. Therefore, the responses might be biased, e.g., social desirability, recall bias. Finally, we did not differentiate between children with overweight and obesity. It is possible that associations between BMI SDS and eating behavior differ depending on the extent of overweight. Future longitudinal studies with a larger and more representative sample, in which different weight groups can be distinguished, would be useful to better understand the interaction between lifestyle and weight development.

## Conclusions

The present analysis showed that an unhealthy diet and eating routines as well as a lack of PA are associated with overweight, including obesity, in children and adolescents. The associations with an unhealthy diet could mainly be observed in families with medium SES. In children from families with low or high SES, other factors besides eating behavior might play an essential role in the development or maintenance of overweight.

## Data Availability

The legal requirements and the given informed consent do not allow public sharing of the dataset. Interested researchers can contact the research data management of the Medical Faculty, University Leipzig: forschungsdaten@medizin.uni-leipzig.de for further information. The dataset ID is PID-00080/01.

## Abbreviations

BMI: Body Mass Index
CoCu: Composition and Culture of eating
NHS: Nutritional Health Score
OR: Odds Ratio
PA: Physical activity
SDS: Standard deviation score
SES: Socio-economic status
